# Spontaneous Splenic Artery Pseudoaneurysm Rupture Causing Hemorrhagic Shock

**DOI:** 10.7759/cureus.8286

**Published:** 2020-05-26

**Authors:** Tim Montrief, Mehruba Anwar Parris, Jonathan S Auerbach, Jeffrey M Scott, Jorge Cabrera

**Affiliations:** 1 Critical Care Medicine, University of Pittsburgh Medical Center, Pittsburgh, USA; 2 Emergency Medicine, Jackson Memorial Hospital, Miami, USA; 3 Pulmonary and Critical Care Medicine, University of Miami Miller School of Medicine, Miami, USA; 4 Cardiothoracic and Transplant Critical Care, Miami Transplant Institute, Miami, USA

**Keywords:** pseudoaneurysm, hemorrhage, imaging, retroperitoneum, angiography, splenic trauma, pseudoaneurysm of splenic artery

## Abstract

Splenic artery pseudoaneurysm (SAP) is an uncommon etiology of acute abdominal pain, requiring a high degree of clinical suspicion to diagnose in a timely manner. There are currently no reports of spontaneous SAP ruptures in the emergency medicine literature. We report a case of a man who presented with acute abdominal pain secondary to an SAP. A computed tomography angiography scan of the abdomen revealed a ruptured SAP with hemoperitoneum. He successfully underwent emergency laparotomy and surgical ligation of his SAP with splenectomy. SAP rupture remains an under-recognized etiology of abdominal pain, even though it is the most frequent type of visceral pseudoaneurysm. Our case herein reinforces the importance of a broad list of differential diagnoses in the patient with acute abdominal pain, as well as the importance of the emergency physician in identifying an emergent condition and then directing the initial stabilization, resuscitation, and management.

## Introduction

Splenic artery pseudoaneurysm (SAP) is a rare entity, with less than 250 cases reported in the literature, but critical to recognize in the acutely ill patient [1,2]. Patients with SAP may present with non-specific symptoms, including abdominal pain, flank pain, chest pain, nausea, vomiting, or gastrointestinal bleeding [1]. SAP may be an incidental finding; however, the majority of patients present with hemodynamic instability due to SAP rupture [1].

The most common presentation of SAP is gastrointestinal bleeding, which typically occurs from the SAP hemorrhaging into the gastrointestinal tract via the pancreatic duct [1]. Acute abdominal pain occurs in approximately 30% of patients with SAP, requiring a high degree of suspicion for an early and accurate diagnosis [1]. Prompt detection and intervention is critical as untreated SAP rupture has a 90% mortality [3,4]. We present one case of spontaneous SAP rupture presenting as acute left upper quadrant abdominal pain, which was successfully treated with exploratory laparotomy and splenectomy.

## Case presentation

A 36-year-old Hispanic man presented to the emergency department complaining of acute-onset, progressive, sharp, stabbing, colicky left upper quadrant abdominal pain radiating to his left flank that began one hour prior to evaluation. The patient denied any recent trauma but endorsed a history of previous open laparotomy and bowel resection after suffering a stab wound in his left-upper quadrant six years prior.

On physical examination, he was uncomfortable appearing and in moderate distress secondary to pain. His temperature was 36.8°C, blood pressure 110/90 mmHg, heart rate of 114 beats per minute, respiratory rate of 18 respirations per minute, and oxygen saturation 98% on room air. He had warm extremities and equal pulses in his bilateral upper extremities. His abdomen was firm, non-distended, and diffusely tender to palpation with voluntary guarding.

**Figure 1:**
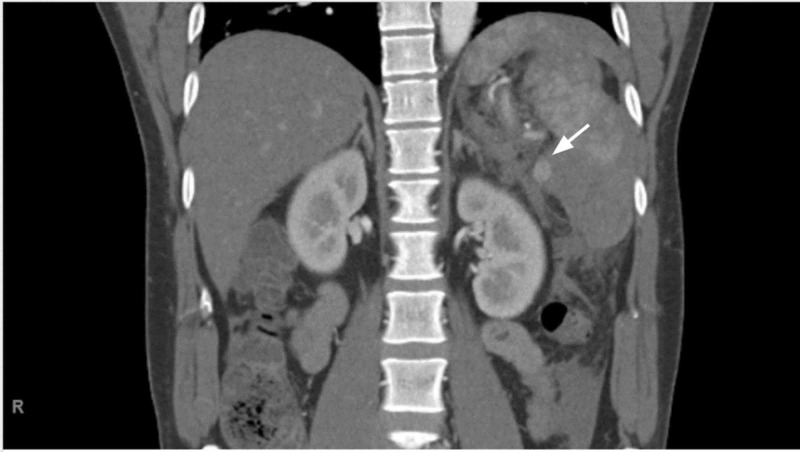
Coronal view of splenic artery pseudoaneurysm There is a round area of enhancement measuring 1.3 x 1.2 cm seen at the posteromedial aspect of the hematoma consistent with splenic artery pseudoaneurysm (white arrow).

**Figure 2:**
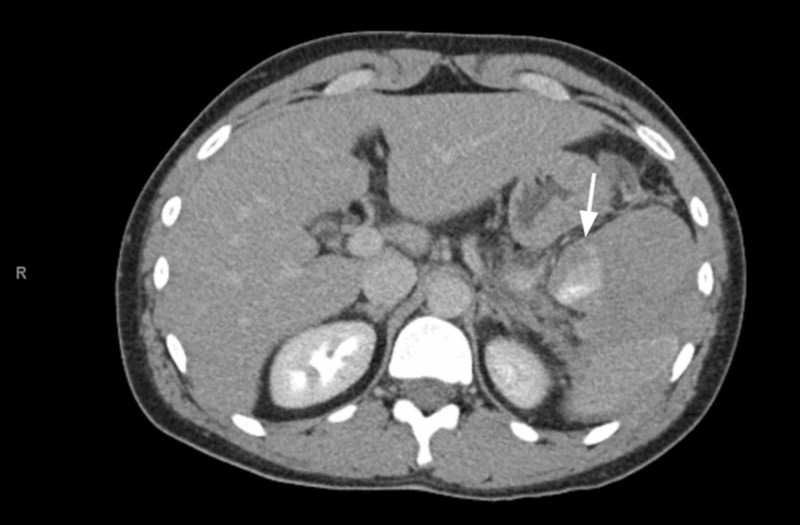
Axial view of splenic artery pseudoaneurysm There is a round area of enhancement measuring 1.5 x 2.7 cm seen at the posteromedial aspect of the hematoma consistent with a bleeding splenic artery pseudoaneurysm (white arrow)

Bedside focused assessment with sonography in trauma (FAST) exam was positive for intra-abdominal free fluid and a large hematoma in the left-upper quadrant. He had a normocytic anemia, with a hemoglobin of 12.1 g/dL, a hematocrit of 35.3%, and lactic acid of 2.3 mmol/L, while all other labs, including lipase, were within normal limits. The patient was resuscitated with two liters of normal saline and underwent a computed tomography (CT) angiography of his abdomen and pelvis. It revealed a 6.8 x 5.1 x 6.6 cm splenic hematoma with an actively extravasating 1.3 x 1.2 cm SAP, hemoperitoneum, and adjacent mesenteric stranding in the adjacent bowel (Figures 1, 2).

The patient was taken to operating room for a laparotomy where intraoperative findings showed two liters of intra-abdominal blood posterior to the spleen, an actively bleeding SAP, and an atraumatic spleen. The splenic pedicle and vein were ligated, and splenectomy was performed. The patient continued to have hemodynamic instability in the immediate postoperative period requiring transfusion of one unit of fresh-frozen plasma and two units of packed red blood cells. He underwent a repeat exploratory laparotomy on postoperative day 1 that revealed active bleeding in a branch of the inferior transverse pancreatic vessels, which was repaired. The patient had an uneventful six-day stay in the intensive care unit before discharge. The patient had inoculation for splenectomy, was started on penicillin, and followed as an outpatient in the surgical clinic with no further complications noted during a two-year period.

## Discussion

SAP is an infrequent, but highly morbid, etiology of acute abdominal pain, and requires a high index of suspicion for a timely diagnosis. While there remains a dearth of cases in the emergency medicine literature, SAP is most commonly caused by acute or chronic pancreatitis, trauma, peptic ulcer disease, or intraoperative injuries [1]. Visceral pseudoaneurysms, including SAP, are at higher risk for spontaneous rupture compared to true arterial aneurysms due to their thin wall. 

The mechanism for the SAP in our patient's case is thought to be secondary to his initial stab wound six years prior, or as an iatrogenic complication of his exploratory laparotomy. As opposed to our patient’s most likely etiology, SAP formation secondary to blunt deceleration injury is more common [5]. During deceleration, the arterial wall is disrupted, and blood collects between the tunica media and tunica adventitia, forming a pseudoaneurysm [6]. Because non-operative therapy of blunt splenic injuries has become more common, it is possible that there will be an increasing incidence of SAP [7]. This is compounded by the fact that time from original injury until diagnosis of pseudoaneurysm varies greatly, from one hour to five years (median <24 hours) according to one study [1].

Within the literature, hemorrhage is the most common finding in patients with SAP presenting to the emergency department, with 14.8% of patients presenting with hematemesis, 26.2% with hematochezia or melena, and 20.3% with evidence of bleeding into the pancreatic duct. Of these patients, 58% were hemodynamically unstable on initial presentation. Approximately 30% of all patients with SAP present with acute abdominal pain. CT angiography and ultrasound are the most common imaging modalities used to confirm the diagnosis in the ED [1]. However, there have been no studies investigating performance of these modalities for SAP, and there are reports of small SAPs being misclassified as peripancreatic fluid collections [8]. Ultrasound is widely available, cost effective compared to CT, and limits exposure to ionizing radiation; however, this modality is limited by bowel gas, arteriosclerosis, and body habitus. In contrast, CT angiography provides information regarding the size, shape, position, and contents of the SAP [9]. CT angiography evaluates the other intra-abdominal organs, investigating other potential etiologies of the patient’s presentation.

Various interventions are available for both ruptured and intact pseudoaneurysms, including splenectomy, SAP ligation, and transcatheter embolization. However, within the literature, a significant number (14%) of transcatheter embolizations are unsuccessful, requiring subsequent surgical intervention. SAP size is not an accurate determinant of rupture, as some of the smallest (0.3 cm) and largest (17 cm) SAPs in the literature have ruptured, although this may be subject to publication bias and no high-level studies have investigated asymptomatic pseudoaneurysms [1].

There is only one other reported case of unprovoked, spontaneous SAP rupture to date, in a critically ill patient who presented with altered mental status, abdominal pain and hemorrhagic shock [4]. He was successfully treated with arterial coiling and embolization. Our case is unique because he presented with hemorrhagic shock from an atraumatic SAP rupture, most likely from iatrogenic injury during his exploratory laparotomy six years prior.

This patient’s insidious presentation combined with SAP rupture’s rarity reinforces the importance of a broad list of differential diagnoses in the patient with acute abdominal pain, as well as the importance of the emergency physician in identifying an emergent condition and then directing the initial stabilization, resuscitation, and management. The patient’s profound abdominal tenderness and history of known exploratory laparotomy and bowel resection initially raised the possibility of an early small bowel obstruction. However, a wide differential included other intra-abdominal infections (i.e., colitis, intra-abdominal abscess, inflammatory bowel disease, appendicitis, or viscus perforation), nephrolithiasis, pulmonary embolism, acute pancreatitis, and mesenteric ischemia. Given the patient’s normocytic anemia and continued pain despite opiate administration, finding a source for presumed hemorrhage was essential to clarifying the etiology of his symptoms. The patient did not have any signs or symptoms of gastrointestinal bleeding; therefore, a point of care ultrasound was performed, which demonstrated intra-abdominal hemorrhage and a hematoma in the left upper quadrant. In the absence of clear gastrointestinal or intra-abdominal bleeding, clinicians should consider occult sepsis, hemolysis, bone marrow failure, renal failure, membrane defects, and enzyme deficiencies as the underlying etiologies of anemia [10].

## Conclusions

Hemorrhagic shock caused by SAP rupture without evidence of gastrointestinal bleeding is uncommon but critical to accurately diagnose and manage, given the high mortality rate if left untreated. Our patient’s presentation highlights the importance of a broad list of differential diagnoses in the patient with acute abdominal pain, as well as a structured approach to the initial evaluation and aggressive management of patients with hemorrhagic shock. As in our patient’s case, multidisciplinary involvement, including interventional radiology and surgical consultation, should be sought early in cases of ruptured SAP.

## References

[1] Tessier DJ, Stone WM, Fowl RJ (2003). Clinical features and management of splenic artery pseudoaneurysm: case series and cumulative review of literature. J Vasc Surg.

[2] Nicaise N, Golzarian J, van Gansbeke D, Cremer M, Struyven J, Deviere J (1998). Rupture of pseudoaneurysm: a cause of delayed hemorrhage after endoscopic cystoenterostomy; angiographic diagnosis and treatment. Gastrointest Endosc.

[3] Huang IH, Zuckerman DA, Matthews JB (2004). Occlusion of a giant splenic artery pseudoaneurysm with percutaneous thrombin-collagen injection. J Vasc Surg.

[4] Schatz RA, Schabel S, Rockey DC (2015). Idiopathic splenic artery pseudoaneurysm rupture as an uncommon cause of hemorrhagic shock. J Investig Med High Impact Case Rep.

[5] Sugg SL, Gerndt SJ, Hamilton BJ, Francis IR, Taheri PA, Rodriguez JL (1995). Pseudoaneurysms of the intraparenchymal splenic artery after blunt abdominal trauma: a complication of nonoperative therapy and its management. J Trauma.

[6] Norotsky MC, Rogers FB, Shackford SR (1995). Delayed presentation of splenic artery pseudoaneurysms following blunt abdominal trauma: case reports. J Trauma.

[7] Davis KA, Fabian TC, Croce MA (1998). Improved success in nonoperative management of blunt splenic injuries: embolization of splenic artery pseudoaneurysms. J Trauma.

[8] Savastano S, Feltrin GP, Antonio T, Miotto D, Chiesura-Corona M, Castellan L (1993). Arterial complications of pancreatitis: diagnostic and therapeutic role of radiology. Pancreas.

[9] Wang CX, Guo SL, Han LN (2016). Computed tomography angiography in diagnosis and treatment of splenic artery aneurysm. Chin Med J.

[10] Ong B, Rockey DC (2014). The syndrome of a large drop in hematocrit in hospitalized patients: clinical features and gastrointestinal bleeding outcomes. J Investig Med.

